# Conflict Between Direct Experience and Research-Based Evidence Is a Key Challenge to Evidence-Based Respiratory Medicine on British Racing Yards

**DOI:** 10.3389/fvets.2020.00266

**Published:** 2020-05-27

**Authors:** Tierney Kinnison, Jacqueline M. Cardwell

**Affiliations:** Royal Veterinary College (RVC), London, United Kingdom

**Keywords:** Inflammatory Airway Disease, equine asthma, evidence-based veterinary medicine, racehorse, veterinarian, qualitative, implementation research, antimicrobial stewardship

## Abstract

Inflammatory airway disease (IAD) is a commonly diagnosed but variably defined syndrome of equine lower airway inflammation. The most recent American College of Veterinary Internal Medicine (ACVIM) consensus statement, informed by research evidence, recommends a case definition based on clinical signs (poor performance or occasional coughing of at least 3 weeks duration), increased endoscopically-visible tracheal mucus, and bronchoalveolar lavage cytology, and proposes that the condition should be termed ‘mild-moderate equine asthma' (mEA). In British Thoroughbred racehorses, research to date has focused on airway inflammation defined by increased tracheal mucus and inflammatory tracheal wash sample cytology. It has been unclear whether or to what extent the ACVIM consensus statement has influenced the practice of British racing veterinarians. The aim of this qualitative study was to characterize and understand rationales for current practices relating to diagnosing and managing airway inflammation in British racehorses. Audio-recorded focus group discussions were conducted with 25 participants from four veterinary practices in England. Practices were purposively selected to represent those responsible for different types of racehorse, in different geographical regions. Thematic analysis of transcripts identified (i) an over-arching theme of *serving the racing industry* within which two further themes (ii) *disregarding of the consensus* and (iii) *the pragmatic clinician* were nested. The requirement to serve the racing industry was a key driver of clinical approaches, strongly influenced in particular by the trainer. Participants widely disregarded the consensus case definition of IAD/mEA for British racehorses because of perceived differences in etiology, perceived lack of practicability, particularly of BAL sampling, and perceived lack of understanding of the British racing context by consensus authors. Participants shared a strong professional identity as pragmatic clinicians providing an individualized clinical approach based on direct experience, which was often prioritized as the most valuable evidence with which to inform clinical decision-making. Lack of alignment with international consensus presents a barrier to practicing and furthering evidence-based medicine. Improved dialogue and partnership in research would be valuable and further research tailored for this population, including continuing development of contextually acceptable diagnostic methods, may be required.

## Introduction

Inflammatory Airway Disease (IAD) is a commonly diagnosed but, historically, variably defined syndrome of equine lower airway inflammation. It is the most common form of respiratory disease in British Thoroughbred racehorses (1–3) and an important cause of reduced performance and days lost to training and racing, particularly in 2 year olds in training for flat racing (4–6). It is less common in older racehorses in training for National Hunt (jump) racing (2, 3, 7), suggesting an infectious etiology in this population, although elsewhere evidence suggests that it is primarily non-infectious [e.g., (8–10)].

Terminology has varied over the years and a variety of case definitions and assessment methods have been reported (1, 3, 11–18). Much research has focused on cases defined partly by bronchoalveolar lavage (BAL) sampling, used routinely for respiratory assessment in many equine populations [e.g., (9, 12, 19–22)] including at the racetrack (20, 22). The latest American College of Veterinary Internal Medicine (ACVIM) consensus statement recommends a case definition based on clinical signs of poor performance or coughing of at least 3 weeks duration, in the absence of fever or other systemic signs, in combination with excess endoscopically-visible tracheal mucus or inflammatory BAL cytology (23). It was also proposed that the condition should be termed ‘mild-moderate equine asthma' (mEA), distinguished from severe equine asthma (recurrent airway obstruction) by the absence of increased respiratory effort at rest.

Elsewhere, particularly in the United Kingdom, research on racehorse airway inflammation has centered on increased endoscopically-visible tracheal mucus, coughing or increased inflammatory cells in tracheal wash (TW) samples [e.g., (1–3, 7, 8, 11, 14, 15, 17, 18, 24, 25)]. On British racing yards, tracheal endoscopy have been the basis of lower airway diagnostic investigations for many years and, anecdotally, routine endoscopic examination of young racehorses, often including those that are outwardly healthy, remains common practice. As the clinical significance of increased tracheal mucus in the absence of either clinical signs (e.g., chronic coughing, poor performance, or exercise intolerance) or BAL-based evidence of inflammation, is not clear, current consensus opinion is that this approach is likely to result in some horses being misclassified as IAD/mEA-affected. While there is some evidence that increased tracheal mucus is associated with poor performance (26–28), tracheal mucus alone is not considered to be a sufficiently reliable indicator of the IAD/mEA phenotype (23). Application of consensus-recommended diagnostic criteria means that the syndrome of increased tracheal mucus and inflammation detected in TW samples, commonly observed in young British TB racehorses in training, cannot be defined as IAD/mEA.

Given the anecdotal evidence that tracheal-based sampling remains standard practice on British racing yards, the aim of this study was to understand British racing veterinarians' rationales for current approaches to diagnosing racehorse lower airway inflammation. This required a qualitative research approach, which allows in-depth exploration of views, perceptions, and experiences underpinning rationales. Objectives were to characterize veterinarians' opinions and perceptions, and to understand the challenges they face, in relation to practicing evidence-based respiratory medicine.

## Materials and Methods

Ethical approval for the study was obtained from the Royal Veterinary College Social Science Research and Ethics Review Board (URN: SR2017-1224). Qualitative data were gathered through semi-structured focus group discussions. A topic guide was designed to capture current practices and opinions relating to the nature, diagnosis and management of lower airway inflammation, as well as familiarity with, and views on, the most recent IAD consensus statement. Focus group discussions were led by author TK, a non-veterinary researcher experienced in qualitative methods. Participation was voluntary and consent was obtained individually. Participants were first asked ‘What is IAD?,' followed by questions about diagnostic and treatment decisions, and were provided with an excerpt from the latest ACVIM consensus statement on IAD to guide discussions about its implementation.

Four veterinary practices were purposively selected to represent contexts in which opinions were likely to differ, such as those responsible for different types of racehorses, including young (primarily 2-year old) racehorses in training for flat racing and older (>3 year old) racehorses in training for National Hunt (jump) racing, in different regions of England. Focus groups were arranged by TK via phone or email and held on practice premises between December 2017 and March 2018. Discussions were audio recorded and recordings were submitted to an external company for verbatim transcription. Transcriptions were assessed for accuracy by TK and sent to all participants to allow identification of any inaccuracies.

An inductive thematic analysis was conducted on all transcripts by author TK, based on the method described by Braun and Clarke (29), from a critical realist stance—i.e., seeking to understand experiences, perspectives and social influences rather than an entirely objective ‘reality.' Analysis comprised repeated reading of the transcripts, with initial semantic coding of data segments (sentences or paragraphs) according to the topic or concept conveyed. Codes were compared between interview transcripts and an iterative process of refining codes (merging or splitting, and moving from description to interpretation) was undertaken. Refined codes were organized into preliminary themes (related concepts) and sub-themes, which were discussed between the authors and further refined to ensure findings were contextually meaningful. A preliminary summary of findings was shared with focus group participants, who were invited to outline any disagreements. Two participants (one each from two of the four groups) confirmed that the summary was representative of their discussions, and no group reported any disagreement.

In the results section, direct quotations are presented to illustrate findings and demonstrate grounding of the analysis in the original data. Respondents are identified by their focus group (FG) number and their individual respondent number (R).

## Results

Focus group characteristics are summarized in Table 1. Group size ranged from 3 to 11 participants. Of the total of 25 participants, five were women. With the exception of two veterinary-qualified (FG1) and one non-veterinary (FG4) laboratory staff members, and one veterinary student (FG3), all were veterinary clinicians, with levels of experience ranging from recent graduates to senior partners. Focus group discussions lasted between 46 and 74 min.

**Table 1:** Summary of focus group characteristics.

**Group**	**Number of participants**	**Male: Female**	**Clinician: Lab**	**Flat/NH**	**Location**	**Duration**
1	6	5:1	4:2	Flat	East England	67 min
2	3	3:0	3:0	Mostly jump	West England	46 min
3	11	9:2	11:0	Mostly Flat	East England	56 min
4	5	3:2	4:1	Mixed	South England	74 min
Total	25	20:5	22:3			Mean: 61 min

*Flat, young racehorses in training for flat racing; NH (National Hunt), older racehorses in training for jump racing*.

**Figure 1:**
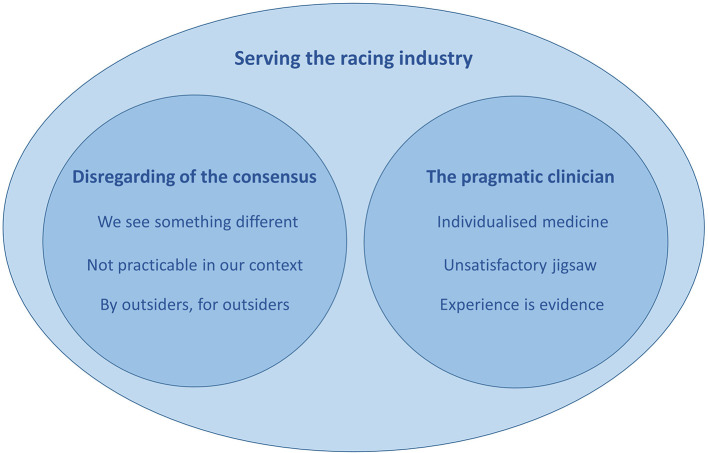
An overview of themes and sub-themes developed through thematic analysis.

Three key themes were developed from analysis of focus group transcripts: (i) An over-arching theme of *serving the racing industry* within which two further themes (ii) *disregarding of the consensus* and (iii) *the pragmatic clinician* were nested. An overview of themes and sub-themes is presented in Figure 1.

### Serving the Racing Industry

It was evident throughout discussions that, for these participants, serving the racing industry was a key driver of clinical approaches to racehorse respiratory health. This was seen in particular through the strong trainer influence on when and how respiratory diseases are diagnosed and treated. The focus of diagnostic investigations for lower airway inflammation is endoscopic assessment of the amount and character of tracheal mucus. At the outset, it is usually the trainer who selects horses for endoscopy, often as a routine health check or because of training or racing plans rather than on the basis of any frank clinical signs such as coughing or poor performance:

*Generally, we don't initiate it. Sometimes we might say, “This horse isn't performing well - shall we scope it?”, but the vast majority of times the trainer has already decided what he wants doing [FG4, R3]*.

*It's a large yard and [the trainer has] a long history of scoping a lot of horses - sometimes yard wide but often just pre-race and pre-declarations [FG1, R2]*.

This means that, on British racing yards, there is no standard or systematic method of monitoring, diagnosing, or managing racehorse respiratory health. Horses being examined endoscopically are not necessarily unhealthy and the scoping of outwardly healthy horses is regarded as proactive:

*The amount of work that goes into investigation will very much depend on the type of horses the particular trainer has and his understanding of the consequences, especially of not doing anything. Some trainers will only get you to look at the horses if they cough and others have got a much more proactive approach [FG3, R6]*.

The trainer's approach to this varies with personality, experience and training methods, general yard health, and stage of the racing season:

*We have some trainers who […] when they're in a rocky patch performance-wise, they won't automatically ring up the vet and say “I think the horses are ill” or look for a problem there. They'll think “Well, actually, it's a worse bunch of 3 year olds this year. We've had bad luck” [FG1, R2]*.

*If a horse coughs once, a week before Royal Ascot, then they'll want it scoped and potentially treated. Or if a horse was coughing now, particularly with some horses this time of year moving from yard to yard and mixing with ages and mixing with populations - a certain amount of coughing is definitely tolerated this time of year [FG1, R1]*.

Veterinary involvement is therefore influenced strongly by trainer expectations, which are in turn driven by owner expectations, finances and other racing-related pressures such as the need to explain a disappointing race or season:

*[The trainer might say] ‘We've come to the races having told the owner “we should be in the placings today”' […] and they're then needing some genuine help in some cases, or an excuse in others [FG2, R2]*.

While the clinicians recognized that the commonly-seen lower airway inflammation would likely resolve without treatment, and with rest, they were under pressure to return horses to optimal health as quickly as possible, because of the requirement for the horses to perform athletically:

*The [young horse] condition that we're talking about is self-limiting and the horses will get over it themselves if you back off … But the owners don't really want to hear that you've backed off […] because they want to get on - which is why we take a more active, aggressive approach [FG3, R10]*.

The pressure on trainers to train and race leads to an expectation of treatment, and in particular of antibiotic treatment. This, therefore, puts pressure on clinicians to prescribe antibiotics, and raises the issue of antibiotic stewardship:

*It's consumer driven - clinical signs are really the only thing that the trainer or the owners are concerned about and (this is a great generalisation) the quickest way to stop a bunch of horses coughing is to put them on antibiotics [FG1, R1]*.

This was the area of greatest dissent amongst participants. Some clinicians described antibiotics as the first line of defense (“*by and large they will get a course of antibiotics before anything else” [FG1, R1]*), while others would often, or usually, perform culture and sensitivity testing before making treatment decisions:

*I can't say that every horse that we would put on antibiotic medication would have had a culture and sensitivity. A reasonable percentage would, but we would be basing it on knowing what was active in the yard and what horses had responded to what medication. [FG2, R2]*.

*Antibiotics will be based on bacteriology - I won't be using any antibiotics if I have no growth. And then whilst potentially sulphonamides and oxytetracyclines would [usually be] the primary drugs, it will be based on sensitivity [FG4, R1]*.

The issue of antibiotic stewardship was recognized and discussed, as seen through the lens of serving the racing industry:

*[Antibiotic resistance is] not a good thing and we do see it more and more. So we may have to look at what we do, especially when you lose one or two antibiotics for a period of time – you suddenly realise there's actually very few left in the armoury and we probably need to look after them a bit more [FG3, R1]*.

Overall, pressures and constraints relating to the need to serve the racing industry were strong drivers that underpinned many topics discussed in all groups and will continue to be apparent in the next two themes.

### Disregarding of the Consensus

Participants unanimously disregarded the consensus case definition of IAD/mEA for application to British racehorses. Firmly-held opinions, based on collective experience, were that a different clinical presentation is most commonly seen in this population and that the diagnostic methods required by the consensus definition are not practicable in this setting. Overall, the consensus was seen as something devised for others, outside of British racing, by others or outsiders without relevant knowledge, understanding, or experience.

#### We See Something Different

Although participants agreed that elements of the consensus case definition, namely cough, increased tracheal mucus and poor performance, were relevant to them, the case definition as a whole was not seen to relate to the form of lower airway inflammation most commonly encountered in British racehorses, particularly young racehorses. This condition was generally referred to as a ‘dirty scope,' defined on the basis of endoscopic observation of increased tracheal mucus:

*We see predominantly short-lived, 1-3 week long episodes of increased tracheal mucus, with or without coughing, usually with no haematological abnormalities, sometimes preceded by small indicators of illness like [increased] temperature or snotty nose or whatever, and they seem to be mostly self-limiting [FG1, R2]*.

In contrast to the primarily non-infectious etiology of IAD/mEA posited by international consensus, participants widely attributed the British racehorse condition to bacterial infections, partly based on experience of temporal disease patterns in the population:

*There's a definite pattern you can see almost through the Thoroughbred's life and through the different stages of the season or year. So taking yearlings, for example […] you get yearlings from Ireland, some from America, some from England, and they're all coming together and it's like kids at nursery school, the bugs coming together. They've got relatively naïve immunities and they're all going to get exposed to these new bugs [FG3, R4]*.

*We see less and less bacterial growth as they get older. They can be filthy and not cough much and have very little bacterial growth, so… they're moving away from a perceived infectious aetiology to maybe an environmental [FG3, R6]*.

There were, however, some dissenting voices on the issue of etiology. Not everyone was certain that bacterial infections were causal, or the only important aetiological factor:

*I'm sure these organisms are important in disease. What I'm not clear about is where in the pathogenesis - [ie] whether they are a primary cause or whether they are opportunistic pathogens […] I'm not saying they're not important but at what point are they, do they become important? [FG4, R2]*.

*I'm a strong believer that environmental agents are strong factors in its development. I don't believe it's just infectious- environmental factors have a huge role to play [FG4, R1]*.

It was also acknowledged that a minority of cases would persist or recur repeatedly and it is those few that participants regarded as being potential cases of IAD/mEA by the consensus definition:

*One to two horses a year are always dirty when they're scoped, maybe with the odd scope clean or relatively clean, and so if they're the IAD horses, they're a tiny little subset [FG1, R2]*.

*If I had to put a figure on the incidence of [the consensus] definition of IAD in a flat racing yard in [this area of the UK], it would be 1-2 horses in 100 [FG1, R2]*.

The consensus-proposed new terminology was also considered to be problematic, in particular because of the term ‘asthma' being perceived as associated with allergy, and because the scope of this was regarded as being too narrow to encompass the condition commonly seen on British racing yards:

*We're lumping it all under ‘equine asthma' - I think Inflammatory Airway Disease is completely different from allergic airway disease we see in the non-racehorse [FG4, R1]*.

*I don't like the move to ‘equine asthma' because I think it's too narrow and I like the fact that ‘inflammatory airway disease' can cover multiple aetiologies potentially [FG1, R5]*.

In discussing the causes of airway inflammation, participants tended to refer to opinions or points of view, rather than published evidence. The consensus was something to be challenged, or disregarded, in light of their own context-specific experiences. Overall, the condition commonly seen in British racehorses was viewed as either distinct from that seen elsewhere, or an aetiological variation:

*If this is your definition of IAD then there's the majority of [UK] racehorse increased mucus and/or coughing isn't covered [FG1, R2]*.

*I remember being quite confused years ago, going to a talk in [town] and there was an American point of view put forward quite strongly that suggested IAD never had an infectious cause, or a bacterial cause, and I just left the meeting thinking - well, they're not seeing the same horses as us [FG4, R3]*.

#### Not Practicable in Our Context

Participants were unanimously of the opinion that two specific aspects of the consensus case definition, namely the requirement to wait for chronicity of clinical signs before initiating a clinical investigation, and for BAL rather than TW sampling, were not feasible to implement on British racing yards. Given the pressures of getting and keeping horses race-fit, chronicity would never be reached without investigation and intervention:

*I can't think of many yards that would let coughing go on for greater than three weeks without somebody investigating it [FG2, R1]*.

*You'll get trainers who phone you up and say, “Can you come in, in the next 20 minutes and take a trach wash off this horse? It coughed at exercise this morning” [FG4, R3]*.

Participants were also agreed that the trainers they worked with would regard BAL sampling as too invasive, would not tolerate associated interruptions to training and would not be easily persuaded to use it:

*BALs are wholly impractical in race yards. You've got to sedate the horse, you've got to put some local anaesthetic (or I would) and then you've got to suggest that [the trainers] don't canter the horse for several days afterwards, so the prospect of getting [trainers] to do that in horses who are anywhere near hard exercise is pretty limited [FG4, R2]*.

Further to the expected reluctance of the trainers, the participants themselves were not convinced of the diagnostic value of BAL compared with TW, with which they had much more experience and which they considered to be more appropriate for time-efficient health screening:

*Maybe I haven't been doing enough to see the extra benefits, but […] if I'm going in to scope a batch of 12 or 14 horses, I want to be doing that in less than an hour. I want to be four or five minutes per horse - just enough time to clean and tidy up and move onto the next one [FG2, R2]*.

*I couldn't advocate that we'd get any more information, so for a routine screening, I think you'd have to strongly feel that the extra hassle, for want of a better word, is worth it [FG1, R1]*.

However, some used BAL sampling for the small minority of horses with recurrent or persistent problems, which might fit the consensus case definition of IAD/mEA:

*The only time I've been involved with BALs is when I've had a horse that has just manifestly failed to behave the way I thought it would in response to my clinical findings. It might be one a year. It's really low numbers [FG2, R3]*.

#### By Outsiders, for Outsiders

Overall, the view was that British racehorses are a unique population, in a unique setting, which people not experienced in that setting do not fully understand:

*We have a very unique selection or set of horses here. They're all housed together and the yards are all more or less back to back to each other and they're all very young - yearlings and born in 2016 now - […] and I think they're very highly densely populated, they're never turned out […] Everything that could possibly compromise their respiratory system is happening to these horses [FG1, R1]*.

Participants felt that the consensus was not reached by people with appropriate direct knowledge and experience of racing yards, or of the British racing industry in particular, who were perceived to be academics, or ‘university clinicians' rather than field clinicians:

*The problem is most of these consensus statements are from university clinicians - they're not actually working in the field we're talking about […] I just don't believe they've got enough experience of racing yards to be making that statement, to be honest [FG4, R1]*.

*These academics have no idea of the type of pressure you come under when you are faced with a large yard that is coughing… if they think we're going to do hundreds of BALs - it's just not possible [FG3, R6]*.

Further to the objections to BAL based on practicalities, there was also a strong view that, while BAL is accepted elsewhere, it would not be culturally acceptable on British racing yards:

*I know the French, apparently they seem much happier with [BAL] as an idea but if you were to start suggesting it to British trainers – culturally they're not expecting you to do that [FG1, R6]*.

### The Pragmatic Clinician

In addition to these explicit references to perceived outsiders, it was implicit elsewhere in discussions that participants shared a strong professional identity as pragmatic clinicians. They expressed that, within the context of serving the racing industry, they were providing a flexible, individualized clinical approach, although they also acknowledged that the ‘jigsaw' of accessible diagnostic information was sometimes unsatisfactory or frustrating. Direct personal or collective experience was commonly lauded as the most useful, available evidence on which to base clinical decision-making.

#### Individualized Clinical Approach

Clinical approach to cases varied, often depending on the clinicians' understanding of trainer expectations, with different methods used in different yards according to trainer preference, particularly in relation to inflammation scoring:

*Trainers sometimes want a score on the whole package. We give a score on what we grossly see and then report the cytology findings, but some trainers have been used to a 0 to 10 score, which takes into consideration the gross endoscopy, the turbidity of the sample and then the neutrophil count, and they put the whole lot together and call it a Grade 7 inflammation or a Grade 4 [FG2, R2]*.

*The protocol in that yard is to take a wash and make a score of the turbidity and add that to the score of the visual and then they sometimes grade them differently themselves [FG1, R2]*.

The clinical approach also depended on the clinician's knowledge of the long-term history or idiosyncrasies of individual horses:

*There's a sort of a cut-off at which we consider something to be ‘dirty' – a cumulative of a visual from the endoscopic [examination] and what you get in the [TW] sample, although we sometimes record that and report it differently to the trainers, based on, we know that there are some horses that always scope ‘dirtier' than normal but that's where they sit and we would probably be less harsh with our grading on those [FG1, R6]*.

Participants shared the opinion that the TW sample is valuable in the experienced hands of a clinician with appropriate knowledge of the yard or individual. One-off, snapshot endoscopic examinations or TWs were considered less valuable than repeated, sequential examinations:

*It makes such a difference having sequential results because you know that that horse always hovers around 60% macrophages - no matter what you do to it, it just seems to sit there - and you're happy enough. And then one day it's got 75% and you think “this is different than it usually is” [FG4, R2]*.

This was all viewed generally positively as a flexible individualized approach, built on an informed understanding of the client and the individual animals and providing the best clinical judgement on the case, given the available diagnostic information:

*It's part of the jigsaw - you've got to build up the picture of the general performance of the yard and that individual's performance. Like all these things in all parts of medicine they're part of the jigsaw of your decision-making process […]. You should never be too rigid with it. [FG4, R1]*.

#### Unsatisfactory Jigsaw

Participants did express some frustration with the incompleteness of this diagnostic jigsaw, particularly the degree of uncertainty about what can be concluded from a sometimes conflicting combination of clinical and laboratory findings, all involving some element of subjectivity. This reduced confidence in the ultimate diagnosis, or treatment decisions:

*If you [submit a TW sample for cytological examination] and the cells aren't quite right and [the horse is due to run on] Saturday in a big race and they've just told you it's worked really well - what do you do? [FG4, R2]*.

*We have horses that we scope that are visually dirty that still go and run and win big races – and so maybe they would have won by a greater margin or maybe it's just an individual thing and that horse is coping with it – but there's this big grey area where you've got horses that potentially have disease as defined by the cytological appearance of the lung and yet might not be showing any outward disease at all or any inhibition of performance [FG1, R2]*.

It was also recognized that there was an inherent systematic bias in the selection of horses for examination, for example, resulting from a desire to explain race disappointments, and a lack of any objective measures of lung function and ultimately performance:

*Very rarely are winners scoped […] If you keep looking for something, you'll find something, but the horses that are performing well may have the same issues [FG1, R1]*.

*You do start to lose your, well your confidence, but you've got to keep returning to the fact that they can only run better when they don't have mucus there. Yes some will run fine but they can only run better if it's not there, surely? I think you've got to keep falling back on that position that with mucus or without mucus, you'd rather have them without mucus [FG3, R1]*.

Despite TW being a routine diagnostic tool, lack of standardization, both of clinical sampling and of laboratory processing of the samples, was an additional problem:

*I'm sure you can take a [TW] sample, split it, and send it to two different labs […] you could in theory get completely different conclusions depending on how that lab is preparing their slides [FG4, R5]*.

*There's a big variation in sampling technique. Not just the timing of when it's taken, [or] the intensity that the horse has exercised - it's actually vet dependent as well [FG4, R4]*.

Therefore, although TW sampling remained the mainstay of their diagnostic investigations, participants agreed that TW cytology was not always useful. A flexible approach to investigation based on the trainer's requirements and the available jigsaw of other diagnostic information was needed.

#### Experience Is Evidence

It was apparent during discussions that the most valued evidence on which to base this complex, clinical decision-making was direct personal or collective experience, regardless of contradictions with research-based evidence or the published consensus. This was particularly evident in the clinicians' defense of the TW sample. Despite the reported challenges of interpreting TW cytology, participants remained of the opinion that TW samples are valuable, and better than research suggests:

*The scientists do take a rather dim view of tracheal washes - they feel it's not representative, you're getting pharyngeal contamination. But experience has shown that it does provide useful information that is reflective of a general widespread lung issue [FG3, R1]*.

*I think tracheal washing post-exercise does represent what's going on in the lungs and I say that based on the fact that I have occasionally done BALs and I haven't found that my results from BAL and tracheal washing are different, really [FG4, R1]*.

In fact, given the collective and cumulative experience of tracheal endoscopy and TW sampling over the years, clinical decisions were often based on subjective visual assessment of gross TW samples, without either cytological or bacteriological laboratory examinations:

*I would say as a general rule, [we] don't submit many tracheal samples to the laboratory. We used to do a lot and I think it's from that experience, of doing it a lot, that we often take the view that you could get as much information as you need, most of the time, from a visual of the trachea and/or the turbidity of the wash [FG1, R2]*.

Again there was a clear trainer influence relating to cost-saving and the pressure to optimize health and performance as quickly as possible. Waiting for the results of laboratory testing was perceived as an unnecessary delay if it would not significantly modify the decisions clinicians would make based on experience:

*We have [trainers who] are not really happy with routinely going to the expense of a trach wash and its laboratory costs as well and they will expect us to make some sort of judgement on the basis of - is it just excessive mucus? Is it mucopus? Is it frank pus with blood? I think it's not unreasonable on some of those to make a sort of semi-educated guess about whether this is likely to be just inflammatory or is there an infectious component? [FG2, R3]*.

Laboratory diagnostic work-up was sometimes reserved for those cases that were not responding to initial treatment:

*If they didn't respond to a decent course of initial antibiotics, you might then start to do some laboratory work or more typically put them on a trial of an anti-inflammatory drug and then re-scope them and see if they respond to that. It's trial and error [FG1, R2]*.

This pragmatic, ‘trial and error' approach was driven by the requirements of a heterogeneous group of trainers, the particular characteristics of the population of horses they train (regarded as unique), and the need to rely on personal and collective experience in the absence of suitably-tailored research-based evidence or consensus.

## Discussion

The aim of this study was to understand British racing veterinarians' rationales for current practices relating to diagnosing and managing racehorse lower airway inflammation.

Findings indicate that British racing veterinarians are striving to serve the racing industry within the constraints of culture, expectations, available resources and evidence, incorporating their collective clinical expertise and knowledge of individual racehorses, as well as owners' and trainers' preferences and demands. This is consistent with the Royal College of Veterinary Surgeons description of evidence-based veterinary medicine as requiring incorporation of “clinical expertise, the most relevant and best available scientific evidence, patient circumstances and owners' values' (30).

Findings also highlight a general valuing of personal and collective experience over research-based evidence and consensus, in this specific context of racehorse lower airway inflammation. Reasons for this were both explicit (with participants expressing firm views on a perceived lack of contextual relevance and practicability of the current consensus on defining and diagnosing IAD/mEA) and implicit (with a strong sense of professional identity as pragmatic clinicians possibly contributing to a degree of ‘othering' of expertise from outside this context). These issues result in a lack of alignment with current international consensus, which is an inherent barrier to practicing and furthering optimal evidence-based respiratory medicine in this population of horses.

The strong over-arching theme of ‘serving the racing industry' is not unexpected given that much veterinary work sits within the classic triad of clinician-patient-client, with the veterinarian's behaviors and decision-making driven in part by client needs or demands, which can lead to ethical discord (31). However, in this particular context the nature of the racing industry adds further complexity and potential conflict. Rather than a single client, there is a network of interested parties, including owners, trainers and jockeys, involved with each racehorse's care, training and prowess, and playing a role in influencing clinical decision-making. Furthermore, this network is situated within a multi-million pound industry, driven by the complex challenge of optimizing equine athletic performance, in which there may be very small differences between success and failure, and by the desire to understand or explain disappointing outcomes that might not reflect any underlying pathological process.

In equestrian sport in general, it is argued that promoting evidence-based decisions over economics-driven decisions is an important aspect of a veterinarian's role (32). However, this is not always straightforward. On British training yards, the pressure to keep racehorses in training and the trainer-driven selection of individuals for endoscopic examination mean that the consensus case definition requirement for chronicity of clinical signs (23) is rarely, if ever, met. Horses without frank clinical signs are often examined, reducing the positive predictive value of any subsequent diagnostic investigations. This is further confounded by the reported lack of acceptability, to both trainers and veterinarians, of BAL sampling, leading to reliance on TW sampling, for which evidence of a correlation with lung function is unclear (33, 34). The focus on endoscopically-visible tracheal mucus is supported by some evidence for a relationship between mucus and poor performance (26–28, 35), but there is less evidence for appropriate, non-invasive investigations of etiology and therefore treatment.

The need to serve the racing industry also creates conflicts with best practice in the important area of antibiotic prescribing. As in other contexts, including both farm (36, 37) and companion animal (38) practice, racing veterinarians perceive a pressure to treat their patients according to client demand or expectation. Prompt treatment of racehorses is required to minimize any potential effect on performance or progress in training. Our participants reported that their clients, both trainers and owners, tended to expect antibiotic therapy for the commonly-identified condition of increased tracheal mucus, even in the absence of coughing or other signs, ideally without the delay and added costs of laboratory culture and sensitivity testing. However, in this study we did not capture clients' perspectives, which would require further investigation. Research in small animal medicine identified that while veterinarians felt a pressure from clients to treat, clients sometimes perceived the veterinarians to be overprescribing antibiotics (39). A systematic review of prescribing practices relating to childhood infections in human medicine identified a conflict between the ‘just in case' prescribing of uncertain clinicians and parents' views that antibiotics should be used only when they will relieve symptoms (40). Both medical and veterinary literature therefore suggests that clinicians may need to develop new strategies for advising clients about antibiotics, and for understanding and managing client expectations in this regard, particularly when the requirement for antibiotics is not certain (37, 41).

It seems, however, that it is not only perceived client pressure that drives antibiotic prescribing on British racing yards. There was also a widely-held view amongst participants, based on collective experience, that airway inflammation is commonly caused by bacterial infections, particularly in young (yearling and 2-year old) racehorses. This is supported by published evidence of reducing prevalence of both IAD (defined in most cases by increased tracheal mucus with or without TW neutrophilia) and infections with age and time in training (3, 7, 14, 42) and by participants' experience that many cases respond to antibiotics. It was also acknowledged, however, that cases would likely resolve naturally given time.

Antibiotic stewardship was not a focus of our study, but it has been identified as an area of concern for equine practice (43, 44), and our findings indicate that it warrants further investigation in the context of British racing. Authors of a UK study, which identified that few equine practices had prescribing guidelines, suggested that such guidelines could reduce inappropriate antimicrobial usage (45). However, a recent Australian study reporting variations in dosing and duration of antimicrobial therapy suggested that few equine practitioners with a policy in place identified it as a source of information for their therapy choices (46). Any investigation of antimicrobial usage in the racing industry would benefit from a sensitive exploration of a complex set of challenges, from multiple perspectives.

The latest consensus statement on IAD/mEA does consider the possibility of an infectious etiology for the airway inflammation commonly seen in young racehorses in Britain (23). However, there is not yet sufficient evidence to support a clear understanding of how this condition relates to that defined as IAD/mEA. Overall, the view from our study participants was that what they see is a different condition and that the consensus-proposed case definition lacks relevance in their context. Research evidence that is not, or is perceived not to be, sufficiently tailored to needs, is a barrier to change in any population (47). Trust in the credibility of a source is required to turn any information into usable knowledge, to create ‘buy-in' to methods and to result in positive outcomes (48). Similarly, information from an ‘in-group' is perceived as more useful than that from an ‘out-group' (49). The ‘for outsiders, by outsiders' sub-theme in our analysis highlighted that, from the perspective of British racing veterinarians, the consensus statement does not come from a source sufficiently well-versed in culture and practices on British racing yards. It is seen as coming from outsiders in terms of geography, as well in terms of experience or understanding of the nature of British racing yards, trainers and racehorses, and from academics, rather than field clinicians. The frank expressions of these views, combined with the implied shared professional identity of ‘the pragmatic clinician' among participants, suggested an ‘othering' of consensus authors consistent with social identity theory, with the risk of intergroup bias (50). However, we would argue that this stemmed from context-specific knowledge and experience rather than simply from inherent distrust or animosity toward those outside the context. These observations are in no way intended as a criticism of either the consensus statement or the British racing veterinarians. Whether subjective perception or objective ‘fact' is regarded as the problem, there is nevertheless a barrier here—even within the same profession—to evidence-based progress and practice.

More generally, our findings raise the question of how different types of evidence are valued or prioritized by field clinicians compared with academics. Study participants tended to highlight experiences, views and beliefs that contradicted research-based evidence, or consensus. The classical evidence hierarchy is often depicted as a pyramid, with anecdotal evidence and expert opinion at the base (low value but most common), and increasing levels of quality of research-based evidence ascending toward the peak (higher value but less common). In the absence of research-based evidence perceived to be credible, relevant and practicable, the most valuable evidence available to the pragmatic clinician is their own personal and collective experience. The best research evidence has no impact on health and welfare if it is not implemented. BAL-based equine respiratory research is therefore of little value to this population while BAL remains unacceptable on training yards. We acknowledge that BAL is widely accepted internationally as the diagnostic tool of choice, and is reportedly well-tolerated in other equine populations including racehorses. However, on this matter there was no dissent among our study participants. Endoscopic assessment of tracheal mucus, with or without TW cytology, continues to be the mainstay of lower airway assessment on British racing yards. Given the strength and consistency of the views expressed across our participant groups, it is clear that adoption of BAL sampling would require a substantial paradigm shift, which will not be effected simply by ‘knowledge transfer' or reiteration of the message from elsewhere that BAL is the well-tolerated diagnostic tool of choice. An active, multi-directional exchange of information, or ‘knowledge translation' approach, with collaboration between those generating and those using evidence is required (47). Given the evident considerable influence of trainers in this particular context, research into trainers' opinions, perceptions and rationales would be useful to characterize further and explain this apparent impasse.

In the absence of such a paradigm shift, more research seeking to develop other minimally-invasive field diagnostic tools, such as biomarkers in easily obtainable samples, or portable and suitably sensitive lung function tests, would be valuable for this equine population. Participants' experiences that TW cytology can be difficult to interpret, especially when it conflicts with clinical findings, are entirely consistent with research-based evidence to date. It is the lack of consistent evidence for a relationship between TW cytology and BAL cytology, poor performance, lung pathology, or lung function that excludes TW cytology from the consensus case definition (28, 51, 52). As pragmatic clinicians working within the constraints of serving the racing industry, and with the benefit of considerable direct personal and collective experience, participants in this study defended the use of tracheal sampling as part of their clinical decision-making, but reported a common practice of gross visual examination of the wash sample without further laboratory testing. This has been described by Ramzan et al. (17), although the association with performance is unclear, with just one study reporting no detected associations (26). However, interpretation of all lower airway clinical and diagnostic findings in racehorses is limited by a lack of a readily-available gold standard indicator of lung function, and ultimately performance.

As this was a qualitative study, the sample of participants was purposively selected and necessarily small to allow for collection and analysis of in-depth data. Researchers more familiar with quantitative methods might have concerns about the representativeness or validity of this approach. However, the purpose of qualitative research is not to quantify, but to understand, perceptions, opinions, or rationales. Representativeness is therefore sought by sampling to capture likely diversity, rather than through probability sampling designed to achieve statistical generalisability (53). The required sample size is dependent on the variability in the data obtained, rather than being determined by calculations aiming for a certain precision or statistical power (53). We have captured data from veterinarians working with different types of racehorse, in different locations, with different backgrounds and levels of experience, in the specific context of British racing practice. The considerable homogeneity of views expressed across this heterogeneous sample supports the reliability of the key findings, which highlight substantial challenges to the practice and furthering of evidence-based respiratory medicine on British racing yards.

## Data Availability Statement

The raw data contain candid information of a potentially sensitive nature including individual/group identifiers and participants did not consent to wider dissemination of this. Appropriately anonymized excerpts of the data are presented in the paper and fuller excerpts could be made available on request to the corresponding author.

## Ethics Statement

This study was reviewed and approved by the Social Science Research Ethical Review Board, Royal Veterinary College. The participants provided written informed consent to participate in this study.

## Author Contributions

JC proposed and designed the study. JC and TK co-designed the focus group schedule. TK recruited practices and participants and conducted all focus groups, data coding, and development of preliminary themes. TK and JC worked together to refine and develop final themes and co-wrote the manuscript.

## Conflict of Interest

The authors declare that the research was conducted in the absence of any commercial or financial relationships that could be construed as a potential conflict of interest.
